# Cardioprotective effect of grape polyphenol extract against doxorubicin induced cardiotoxicity

**DOI:** 10.1038/s41598-020-71827-9

**Published:** 2020-09-07

**Authors:** Shynggys Sergazy, Zarina Shulgau, Galina Fedotovskikh, Laura Chulenbayeva, Ayaulym Nurgozhina, Madiyar Nurgaziyev, Elena Krivyh, Yevgeny Kamyshanskiy, Almagul Kushugulova, Alexander Gulyayev, Mohamad Aljofan

**Affiliations:** 1 Private Institution “National Laboratory Astana”, AOE “Nazarbayaev University”, Nur-Sultan, Kazakhstan; 2 RSE “National Center for Biotechnology”, Ministry of Education and Science of Republic of Kazakhstan, Nur-Sultan, Kazakhstan; 3 JSC “National Scientific Medical Center”, Nur-Sultan, Kazakhstan; 4 Khanty-Mansiysk State Medical Academy, Khanty-mansiysk, Russian Federation; 5 Karaganda State Medical University, Karaganda, Kazakhstan; 6 Department of Biomedical Sciences, School of Medicine, AOE “Nazarbayaev University”, Kerey & Zhanibek Khans 5/1, Nur-Sultan, 010000 Republic of Kazakhstan

**Keywords:** Plant sciences, Cardiology, Medical research

## Abstract

Doxorubicin is a chemotherapeutic agent known to cause cardiotoxicity that is thought to be associated with oxidative stress. The aim of the current study is to investigate the role of grape polyphenols’ antioxidant property as cardioprotective against doxorubicin-induced cardiotoxicity. Adult *Wistar* rats weighing 200 ± 20 g were divided into 3 different groups: a doxorubicin group that received a single intraperitoneal administration of doxorubicin (8.0 mg/kg body weight), an experimental group that received doxorubicin and grape polyphenol concentrate (25 mg/kg) via intragastric route, and the third group was a negative control group that received water only. On day 8, blood samples and tissues were harvested for analyses. The results indicated that grape polyphenol concentrate was able to reduce the signs of cardiotoxicity of doxorubicin through the reduction of aspartate aminotransferase activation, increasing the plasma antioxidant levels and decreasing the level of free radicals. The results also showed that grape polyphenol concentrate was able to reverse doxorubicin-induced microscopic myocardial damage. The myocardial protective effect of grape polyphenol might likely be due to the increase in the level and activity of the antioxidant enzymes, superoxide dismutase, catalase, and glutathione peroxidase. In conclusion, grape polyphenol concentrate displayed cardioprotective effect and was able to reverse doxorubicin-induced-cardiomyopathy in experimental rats.

## Introduction

Doxorubicin is an antibiotic commonly used as an antitumor chemotherapy^1,2^. It is thought to achieve its antitumor effect through the inhibition of topoisomerase II and DNA intercalation, which leads to the formation of reactive oxygen species, DNA crosslinking and apoptosis^3^. One of the most serious side effects of doxorubicin is cardiotoxicity, which is commonly observed in doxorubicin-treated cancer surviving patients^4^. While the mechanisms of cardiotoxicity is not yet known, a number of studies claimed that the toxicity is likely related to mitochondrial oxidative stress, p53-mediated mitochondrial-dependent apoptosis, as well as impaired regulation of autophagy^5,6^.

An increase of free radicals and a decrease in the activity of endogenous antioxidants in the myocardium play important roles in the pathogenesis of doxorubicin-induced heart failure. Therefore, there is an urgent need for the development of cardioprotective treatment for doxorubicin-treated cancer survivors. A number of compounds were tested for their potential cardioprotective activity, including polyphenols extracted from medicinal plants and polyphenol-rich-foods^7^.

Polyphenolic compounds have high antioxidant activity including resveratrol, which was shown to have beneficial effects on cardiovascular diseases^8–10^. However, their role in doxorubicin-induced cardiotoxicity is not well understood. Thus, we hypothesized that the antioxidant activity of polyphenolic concentrate may play a role in the prevention or reduction of doxorubicin-induced cardiotoxicity.

## Results

### Doxorubicin-induced oxidative stress and antioxidant effects of polyphenol

Rats treated with doxorubicin showed an increased level of free radicals (measured using D-ROMs) and significantly reduced antioxidant capacity of blood plasma (determined by PAT test), which were reversed in rats treated with grape polyphenol concentrate (experimental group) (Table 1). The level of free oxygen species was the same in doxorubicin treated rats that received grape polyphenols compared to the control group. There was a slight and non-significant decrease in the blood concentration level of antioxidants compared to the negative control, but remained significantly higher than that of doxorubicin only treated rats.

**Table 1:** Antioxidant effects of grape polyphenol concentrate.

The investigated parameters	Healthy animals, n = 6	Doxorubicin, n = 7	Experimental group, n = 9
D-ROMs test, U Carr	367.3 ± 67.2	554.3 ± 35.6*	400.4 ± 14.8^#^
PAT test, U Cor	2,992 ± 414	2,164 ± 172*	2,667 ± 123*^,#^

*P < 0.05 compare to the value in the group of healthy animals.

^#^P < 0.05 compare to the value in the group of control animals. Results presented are mean ± standard error of the mean.

The measurement of aspartate aminotransferase (AST) activity in the blood of healthy rats, doxorubicin rats and the experimental group are shown in Table 2. Compared to negative control animals (healthy rats), doxorubicin treated rats had a significantly increased levels of AST, which indicates myocardial cell damage. It is well known that doxorubicin induced reactive oxygen species (ROS) lead to mitochondria damage, cell necrosis, breakdown of mitochondria, and the release of AST into the blood, which are commonly used as indication of cell cytolysis^11^. Therefore, we can assume that the observed elevation of AST level (40% increase in the doxorubicin treated group) is an example of the development of cardiomyopathy in these animals. Interestingly, compared to the negative control group, which received water only, the AST levels were significantly reduced (20% reduction) in rats that received a single dose of grape polyphenol concentrate, experimental group (Table 2). This is most likely due to the cardioprotective effect of grape polyphenols against doxorubicin induced myocardial tissue damage. Noteworthy, the AST level alone, does not indicate myocardial damage, as it is not entirely produced by the myocardium and it can also be produced by the liver.

**Table 2:** The effect of the polyphenol concentrate on the activity of aspartate aminotransferase, (M ± m).

The investigated parameters	Healthy animals, n = 6	Doxirubicin, n = 7	Experimental group, n = 9
AST, nmol/(s·L)	1,346 ± 82	1,897 ± 168*	1,507 ± 130*^,#^

*P < 0.05 compare to the value in the group of healthy animals.

^#^P < 0.05 compare to the value in the group of control animals. Results presented are mean ± standard error of the mean.

Therefore, to specifically determine the effect on the myocardial, we measured the level of troponin T (TnT), which is one of the most important and commonly used biomarkers for the diagnosis of myocardial damage. An elevated level of TnT indicates cardiac injury. In this experiment we measured the level of TnT in healthy animals compared to doxorubicin only and doxorubicin + polyphenol treated animals (experimental group). The results indicated that doxorubicin treatment had significantly increased the TnT levels in the blood compared to healthy animals, confirming the cardiotoxic effect of doxorubicin (Table 3). While grape polyphenol concentrate was able to reduce ROS levels (compared to doxorubicin group), it failed to significantly reduce the level of serum TnT in the experimental animals.

**Table 3:** Troponin T (TnT).

Sample	ng/ml
Healthy rats	0.153
Dox control rats	1.071
Dox + polyphenol rats	0.808

The results of TnT levels from rat serum. The results are average of 3 independent experiments from 6 different animals.

### Doxorubicin effect on different hematological parameters

The hematological data following doxorubicin administration in both the control and experimental groups are shown in Table 4. In comparison to healthy animals, the doxorubicin treated group showed a significant decrease in the number of leukocytes, including lymphocytes and monocytes, which further confirms the doxorubicin induced systemic cardiovascular stress. Also, doxorubicin administration caused a significant reduction in platelet concentration and hemoglobin levels. While no change in the number of red blood cells was observed, the total concentration of hemoglobin, hematocrit, the average hemoglobin content in a single red blood cell and the average concentration of hemoglobin in the red blood cell in the control group were reduced, another confirmation of the current oxidative stress state.

**Table 4:** Hematological effects of polyphenol concentrate, (M ± m).

The investigated parameters	Healthy animals, n = 6	Control, n = 7	Experimental group, n = 9
White blood cell count, 10^9^/l	15.9 ± 0.70	8.8 ± 0.26*	8.9 ± 0.57*
Lymphocytes, 10^9^/l	10.9 ± 1.19	4.8 ± 0.36*	4.9 ± 0.14*
Monocytes, 10^9^/l	1.2 ± 0.20	0.6 ± 0.07*	0.6 ± 0.04*
Neutrophils, 10^9^/l	3.8 ± 0.50	3.5 ± 0.22	3.4 ± 0.41
Lymphocytes, %	67.3	54.0	54.3
Monocytes, %	7.8	6.3	7.4
Neutrophils, %	25.0	39.8	38.3
Red blood cell count (RBC), 10^12^/L	10.8 ± 0.82	10.9 ± 1.30	10.2 ± 0.94
Hemoglobin (Hb), g/l	203.7 ± 18.74	97.7 ± 11.05*	180.0 ± 19.49^#^
Hematocrit, %	60.7	54.6	58.9
Mean cell volume (MCV), fl	56.5	54.4	53.8
Mean corpuscular hemoglobin (MCH), pg	19.0	17.7	18.2
Mean corpuscular hemoglobin concentration (MCHC), g/l	335.7	305.6	330.4
Red cell distribution width, %	14.8	15.5	15.6
Platelets, 10^9^/l	613.8 ± 39.67	426.3 ± 22.18*	416.8 ± 67.20*
Thrombocrit (PCT), %	0.4	0.3	0.3
PLT, fl	6.7	6.4	6.1
PDW, %	31.0	28.8	28.2

*P < 0.05 compare to the value in the group of healthy animals.

^#^P < 0.05 compare to the value in the group of control animals. Results presented are mean ± standard error of the mean.

### Polyphenol increases the activity and level of SOD

**Figure 1:**
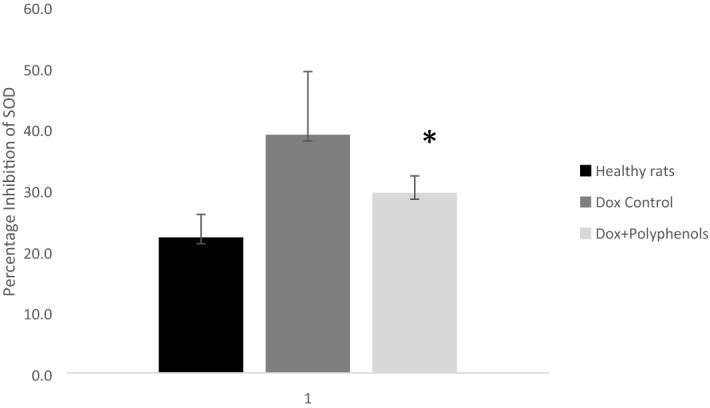
Percentage inhibition of SOD. The graph shows the inhibition of SOD activity as measured by the colorimetric absorbance method. The decrease in SOD activity is proportional to the amount of absorbance at 440 nm. Results presented are mean ± standard error of the mean of 5 independent experiments from 6 different animals per each treatment group. *Represents significance difference (α < 0.05).

**Figure 2:**
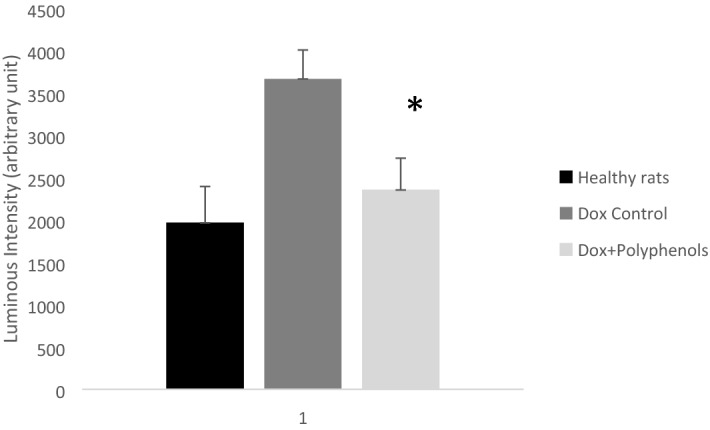
Catalase activity. The graph shows the measurement of hydrogen peroxide substrate remaining after catalase activity. The amount of the remaining substrate is conversely proportional to the antioxidant activity of catalase. Thus, high reading indicates low enzymatic activity. Results presented are mean ± standard error of the mean of 5 independent experiments from 6 different animals per each treatment group. *Represents significance difference (α < 0.05).

There are several enzymes that form a crucial part of the antioxidant defense system including superoxide dismutase (SOD), which catalyzes the dismutation of superoxide into hydrogen peroxide that would then be degraded by catalase or glutathione^12^. The results show that doxorubicin inhibited the activity of SOD, but treatment with polyphenol significantly reversed the inhibition (Fig. 1). The activity and level of SOD are closely associated with other antioxidant enzymes including catalase and glutathione peroxidase. Interestingly, doxorubicin reduced the activity of the antioxidant enzyme catalase and the activity and level of glutathione. However, treatment with grape polyphenol concentrate was able to reverse the inhibition of the activity and level of the enzyme, providing the first possible cardioprotective mechanism of grape polyphenol concentrate (Fig. 2, Table 5).

**Table 5:** Measurement of glutathione level and activity.

Samples	µmol/g protein	Activity (µmol/ml/min)
Healthy rats	3.5 ± 0.3	0.09
Dox control rats	2.4 ± 0.7	0.05
Dox + polyphenol rats	3.3 ± 0.5	0.11

The table shows the approximate glutathione level and activity from 3 different animal groups.

Results presented are mean ± standard error of the mean of at least 3 independent experiments from 6 different animals.

### Electron microscopy

**Figure 3:**
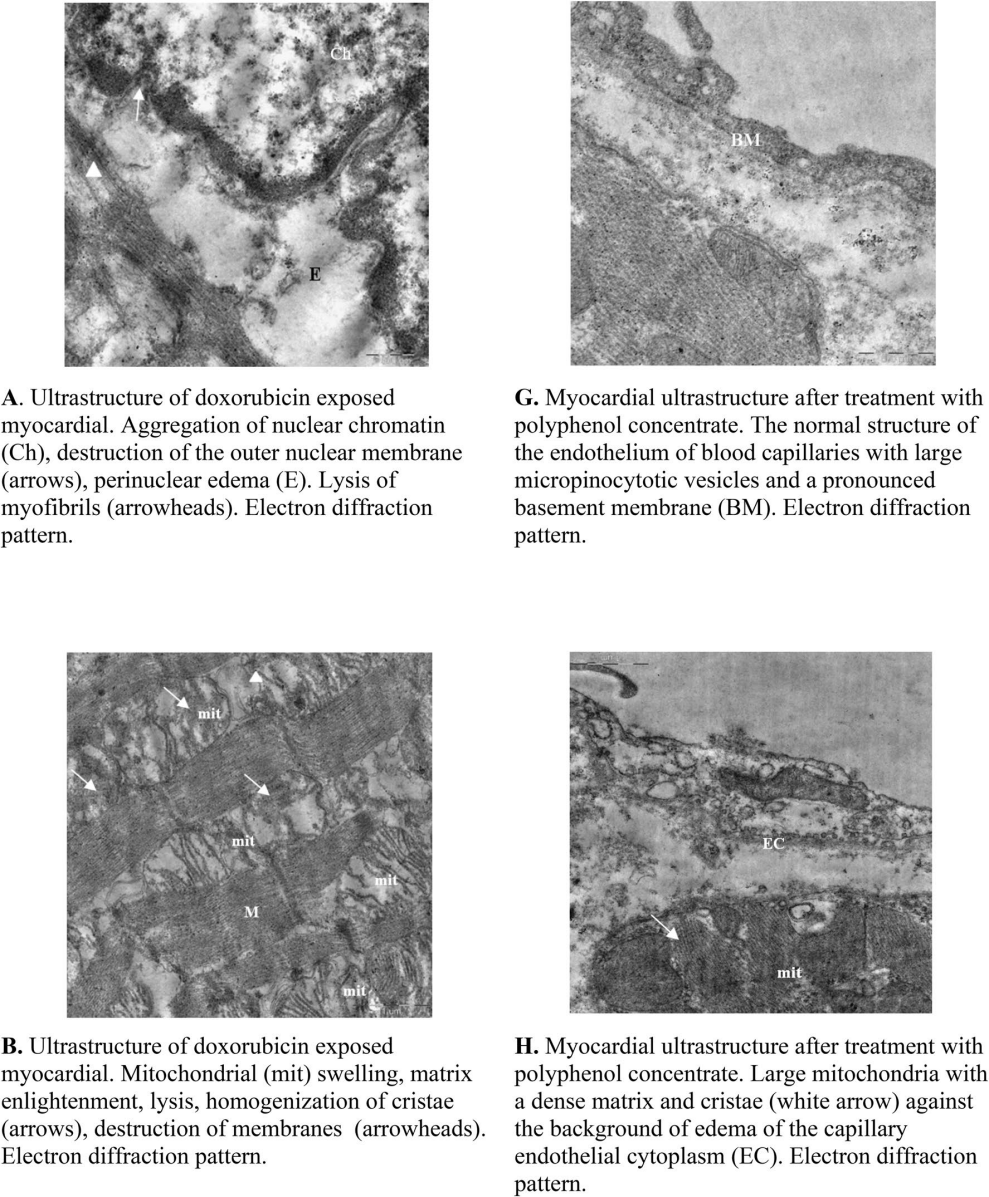

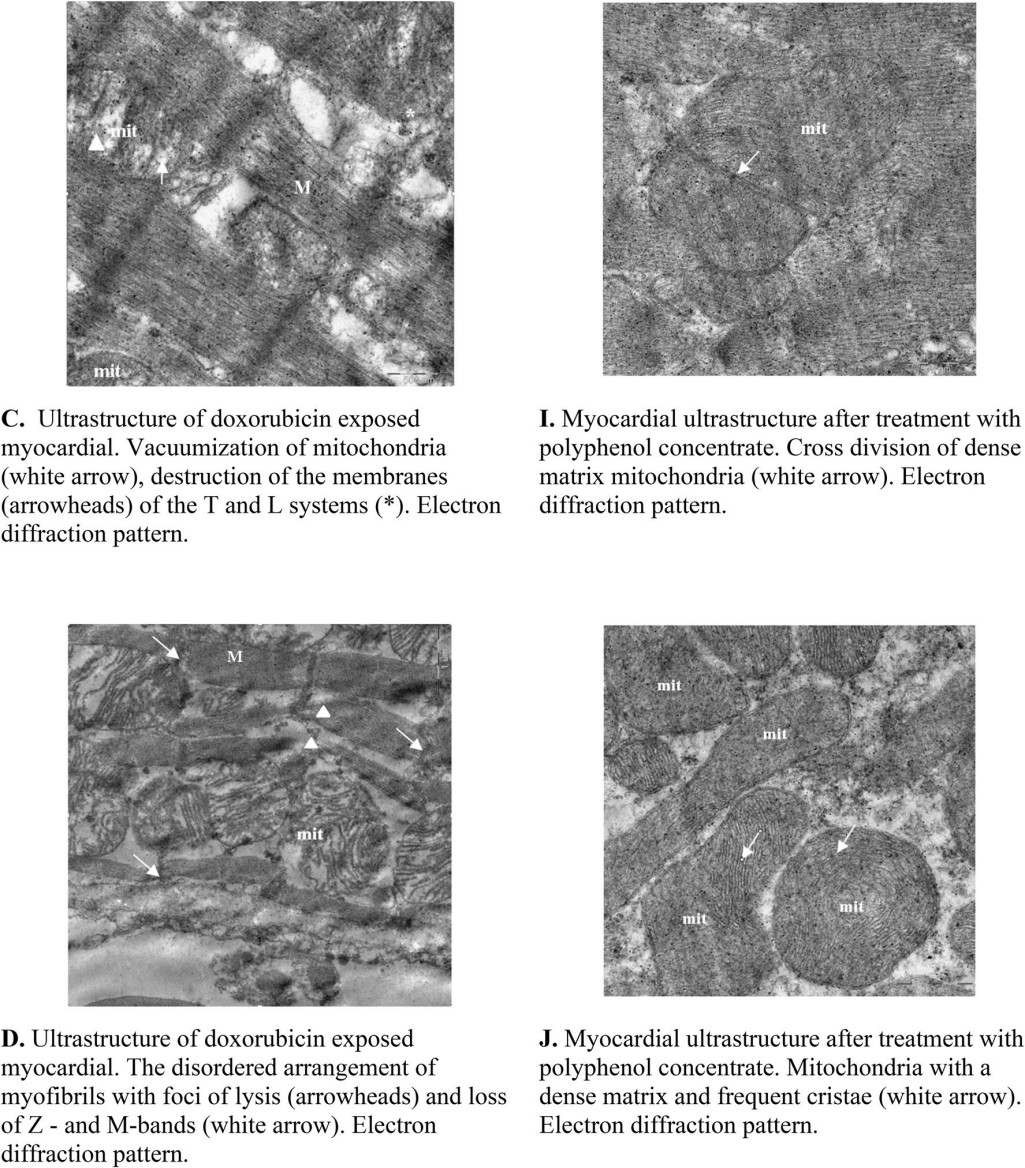

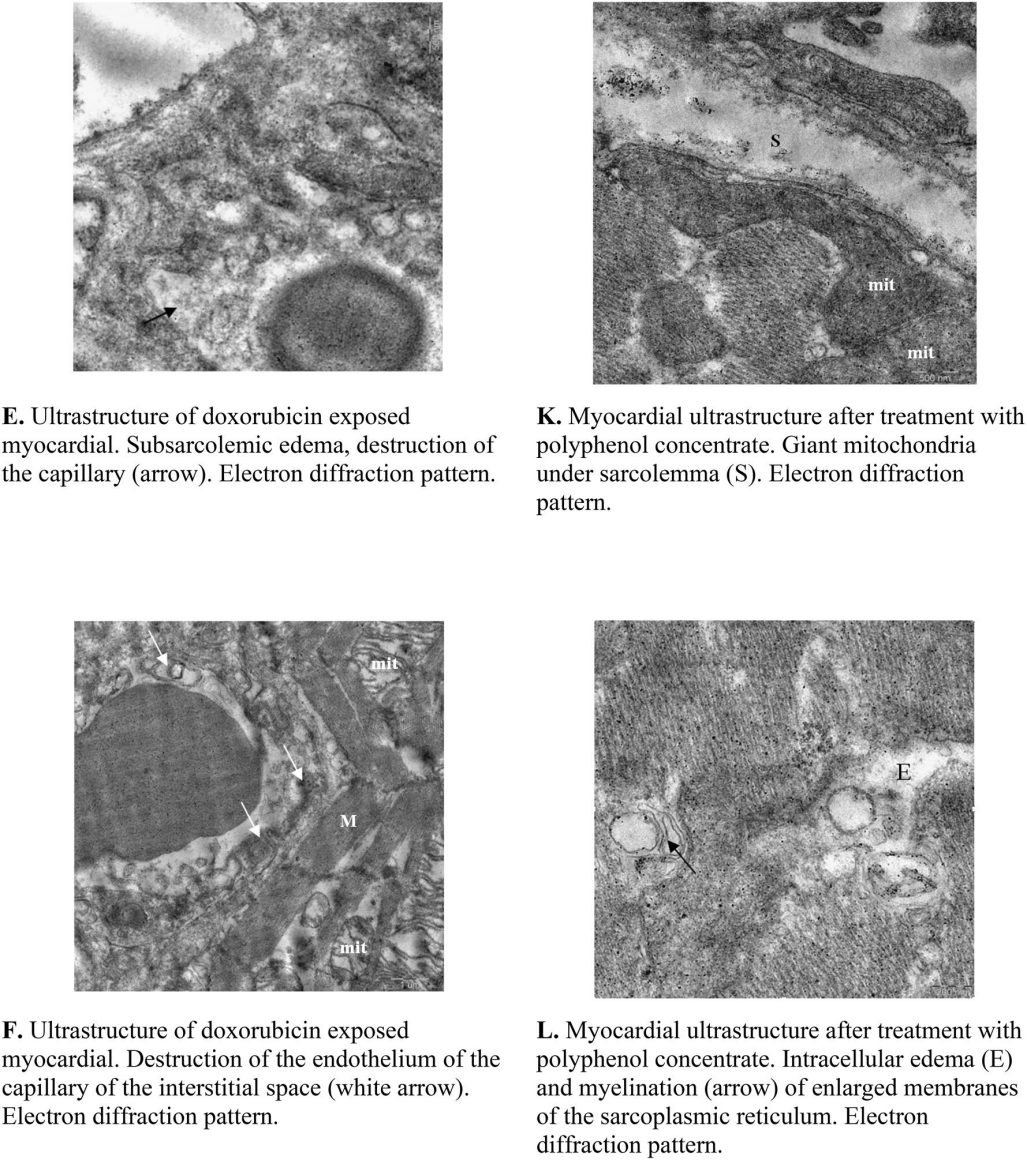
Electron Microscopy analyses. The above images are of rat myocardial tissues from doxorubicin treated animals (**A**–**F**) and grape polyphenol concentrate treated animals (**G**–**L**). Tissues from all the animals (6 per group) were analyzed and the images presented are from at least 3 different animals. Images were captured using MegaView G3 TEM-camera and RADIUS EM-Imaging Software. EMSIS GmbH, Muenster, Germany (www.emsis.eu).

General analyses of electron microscope images of the cardiomyocytes and endothelium from different treatment groups are shown in Table S1 (Supplementary data). The analyses of myocardium from doxorubicin treated rats showed aggregation of nuclear chromatin, dissociation of the outer nuclear membrane and perinuclear edema in the cytoplasm of cardiomyocytes (Fig. 3A). With the exception of single cell necrosis, the ultrastructural changes in the organelles of cardiomyocytes were homogeneous. Oval mitochondria significantly increased in size and were characterized by swelling, sharp enlightenment of the matrix, lysis, homogenization of cristae, destruction and myelination of the outer membranes (Fig. 3B). Individual mitochondria were vacuolated. There was a sharp expansion and destruction of the T and L systems of the sarcoplasmic reticulum and the tubules of the granular endoplasmic reticulum were enlarged and degranulated (Fig. 3C). Also, myofibrils were shown to have a disordered arrangement, varied foci of lysis and loss of Z- and M-bands (Fig. 3D). Other important observations include subsarcolemmal edema, loosening of the sarcolemma and plasma membrane, as well as a decrease in the number of glycogen granules (Fig. 3E). The capillary endothelium of the interstitial space appeared swollen with damaged organelles and a sharp loosening of the basement membrane. Interestingly, some vessels showed complete damage of the lining endothelial as well as materials of the damaged endothelial were visible in the vessel lumina (Fig. 3F).

Interestingly, the results of the electron microscope examination of the myocardium after administration of grape polyphenol concentrate showed a normal endothelial structure of most blood capillaries with the presence of large micropinocytotic vesicles, an abundance of ribosomes and a pronounced basement membrane (Fig. 3G). This indeed confirms the potent antioxidant activity of grape polyphenol concentrate. Intracellular edema in separate capillaries, large elongated mitochondria with a dense matrix and cristae were clearly visible. However, large mitochondria located in cardiomyocytes acquired an irregular lobed shape and were characterized by a matrix of high electron density and densely packed cristae with areas of granular disorganization (Fig. 3H). These giant mitochondria might have formed by the fusion of enlarged mitochondria with adjacent normal sized ones. While the mechanism for the formation of the enlarged mitochondria are unknown, they might have formed as compensation for functional deterioration caused by mutation of mitochondrial DNA^13^. Also, a transverse mitochondrial division was observed (Fig. 3I). Mitochondria appeared to have a concentric arrangement of recovered cristae (Fig. 3J). Also clearly visible was the expanded membranes of the T and L systems, but the subsarcolemmal edema had completely disappeared (Fig. 3K). Interestingly, the treatment with grape polyphenol concentrate resulted in the return of the myofibrils structure back to normal and that glycogen granules were more visibile, which further confirms the potent antioxidant activity of grape polyphenol. However, foci of intracellular edema and myelination of the membranes of the sarcoplasmic reticulum remained visible (Fig. 3L).

### Semiquantitative analyses of electron microscopy

Morphological changes observed in the electron microscopy following the exposure to doxorubicin or grape polyphenol concentrate were assessed semiquantitatively using an adapted semiquantative scoring method^25–28^. The assessments of ultrastructural changes in the cardiomyocytes and endothelial were carried out seperatly and independent of each other. The mean and standard deviation for each of the assessed criteria are shown in Table S2 (supplematry data). The analyses of cardiomyocyte from doxorubicin treated rats, showed significant changes in the shape of the nucleus (Grade 2) with pronounced perinuclear edema, tortuosity of the nuclear membrane (Grade 3) and, in some cases, rupture of the nuclear membrane (Grade 4), as well as some changes in the distribution of chromatin (change in the shape and size of the nucleolus, chromatin aggregation). These changes might not be specific, but were widespread and found in approximately 60% of the examined nuclei. Also, the cardiomycyte of animals that received grape polyphenol concentrate showed evidence of Grade 2–3 damage to the ultrastructures of the nuclei similar to that of the doxirubicin treated group.

Pronounced perinuclear edema (Grade 2) was one of the predominantly observed lesions in the nucleus shape. While the impact of these changes are unknown, changes in the nuclear morphology suggest potential defects in nuclear pore transport and/or transcriptional regulation. Speculatively, the altered shape of the nuclei and nuclear envelopes can induce or amplify changes in the structure of nuclear pores, which will likely affect nuclear cytoplasmic transports and, therefore, the range and functions of the cardiomyocyte nucleus.

The semiquantitative analyses of myofibrils and Z-lines, which are known to affect heart contractility showed: the A- and I-bands, as well as the Z-lines in the I-bands were blurred, in some cases they were thickened or not determined in both treatment groups (Grade 2–3). In some samples, ultrastructural analysis showed signs of sarcolysis and absence of Z-lines (Grade 4). Doxirubicin treated animals showed significant injuries and changes in the structure of myofibrils and some form of dissociation (Grade 2–4), interestingly, these changes were less severe in the grape polyphenol treated animals (Grade 1–2) (µ = 2.8 ± 0.8 vs 1.8 ± 0.4, p = 0.009).

Ultrastructural changes in mitochondria from the doxirubicin only group, displayed all four stages of structural damage (Grade 1–4). Doxirubicin induced various mitochondrial damage including edema, matrix clearing, lysis, homogenization of cristae, destruction with myelination of the outer membranes, and loss of mitochondrial granules, which was reversed by grape polyphenol contracture treatment. It is known that native mitochondrial granules disappear not only under conditions of hypoxia, but also under conditions of high oxygen stress; the restoration of granules in the grape polyphenol concentrate treated group, indicates a high antioxidant activity of the grape polyphenol concentrate that was shown by the reduced level of damage (Grade 1–3). Other important observations include subarcolemal edema, weakening of the sarcolemmal and plasma membrane, as well as a decrease in the number of glycogen granules. Interestingly, a significant increase in the number of glycogen granules was observed in the grape polyphenol concentrate treated animals (p = 0.002).

Morphological analyses of endothelial following doxorubicin treatment showed pronounced edema, damage to organelles, and changes in the basement membrane, as well as in some parts of the vessels-desquamation of the endothelium into the lumen of the vessel. In addition, the nuclei of the endotheliocyte, showed destruction of the outer nuclear membrane, pronounced perinuclear edema, and some changes in the distribution of chromatin (Grade 1–4). These changes were found in about 70% of the observed nuclei in both, doxirubicin only and grape polyphenol contrates treated groups with no significant difference between the two groups (p = 0.937). The antioxidant potency of of grape polyphenol concentrate was evidently shown by its ability to significantly restore the ultrastructures of the mitochondria of endothelial cells in the polyphenol concentrate treated group (p = 0.009), improvement of the general structure of endothelial cells of most blood capillaries (p = 0.041), and the basement membrane (p = 0.026).

The study showed that the toxic effect of doxirubicin causes damage to the ultrastructures of the myocardium. Changes in cardiomyocytes include three main cellular components: mitochondria, nucleus and myofibril structure, and in the endothelium, changes in mitochondria and nucleus. Our results also show that changes in the cytoarchitecture of cardiomyocytes and endothelium caused by the use of doxorubicin can be reduced with the use of grape polyphenol concentrate by returning myofibrils to normal and restoring glycogen granules. Nevertheless, intracellular edema and myelination of the membranes of the sarcoplasmic reticulum persisted in both groups. Consequently, the individual subcellular structural changes observed in this study due to damage from doxorubicin can be partially compensated for by using grape polyphenol concentrate, which may indicate potent antioxidant properties.

## Discussion

In the current study, doxorubicin-induced cardiotoxicity was evidently shown through the increased level of ROS that has resulted in an increased activity of cytolytic enzyme AST and TnT in the blood of treated rats. The damage was also confirmed by the significant changes in the hematological parameters such as the decrease in the number of leukocytes, including lymphocytes and monocytes, and the decrease in the level of platelet concentration. The hematological analyses of doxorubicin treated rats, showed constant red blood cell counts, decrease in the hemoglobin concentration and antioxidant levels, but a significant increase in the level of free radicals. These results are supported by several studies including that of Shinohara et al., which investigated the effect of doxorubicin on blood cells, which reported that doxorubicin oxidizes hemoglobin and membrane protein components 1, 2 and 3 to form a large molecule complex^14^. Furthermore, electron microscopy revealed severe ultrastructural changes in the capillaries of the interstitial space and cardiomyocytes. These lesions were manifested by aggregation of nuclear chromatin, dissociation of the outer nuclear membrane, and perinuclear edema in the cytoplasm of cardiomyocytes. Mitochondria were significantly increased in size and were characterized by swelling, sharp enlightenment of the matrix, lysis, homogenization of cristae, damage and myelination of the outer membranes. The tubules of the granular endoplasmic reticulum were shown to be enlarged and degranulated. Myofibrils had disordered arrangement and varied in foci of lysis. Interestingly, subsarcolemmal edema, loosening of the sarcolemma and plasma membrane were observed, which represent the typical model of doxorubicin-induced cardiomyopathy.

Treatment with grape polyphenol concentrate prevented the doxorubicin reduction of hemoglobin, accumulation of free radicals, as well as the reduction of antioxidant activity in the blood. Speculatively, the treatment with grape polyphenols might have inhibited the doxorubicin-induced oxidization of hemoglobin^14^. Thus, the effect was only seen with hemoglobin, but not the other parameters. The cardioprotective effects of grape polyphenols was further confirmed by electron microscopy analyses of the myocardium, which showed the normalization of the ultrastructure of blood capillaries and cardiomyocytes after the toxic effects of doxorubicin. There was a complete restoration of the structure of mitochondria, disappearance of subsarcolemmal edema, normalization of the myofibrils structure, and clear appearance of glycogen granules, but no effect on the foci of intracellular edema and myelination of the membranes of the sarcoplasmic reticulum.

Nevertheless, doxorubicin-induced cardiomyopathy was reversed following the administration of grape polyphenol concentrate, which was illustrated by the significant increase in the antioxidant activity and the improved hematocrit parameters. Also, animals treated with grape polyphenol showed significant reduction (approximately 20% compared to negative control group) in the levels of AST enzyme, reduction of doxorubicin-induced inhibition of SOD activity, a significant increase in the catalase activity, and an increase in the activity and level of glutathione. Interestingly, cells have different antioxidants to prevent ROS damage, and the most important and indispensable antioxidant enzymes are SOD, catalase and glutathione peroxidase^15,16^. Their collective antioxidant defense cascade starts with SOD converting ROS into H_2_O_2_ and oxygen, and then catalase and peroxidases convert H_2_O_2_ into water, a harmless product^16^. Therefore, based on the current results, we can claim that grape polyphenol achieves its antioxidant mechanism by increasing the levels and activities of these enzymes. This claim aligns with a previous study by Uto-Kondo et al., who showed that tea polyphenol increases endothelium-bound extracellular SOD^17^.

Overall, the use of polyphenol concentrate obtained from Cabernet Sauvignon grapes, reversed the doxorubicin-induced cardiomyopathy through the increase in the total antioxidant activity, limiting the development of oxidative stress, and preventing the cytolytic processes. Thus far, the results in the current study have clearly demonstrated that Cabernet Sauvignon grape polyphenol concentrate can effectively reduce doxorubicin-induced cardiotoxicity, which is likely achieved though their positive association with the potent antioxidant enzymes SOD, catalase and glutathione peroxidase that degrade ROS.

A number of polyphenols are currently being investigated for a possible use as an additional or combinational therapy with antitumor chemotherapy to protect the myocardium from chemotherapy-induced oxidative stress^18–20^. One of the major effects of doxorubicin is that it accumulates in the mitochondria and destroys the electronic chain of mitochondria, which leads to an increase in the production of ROS^21^. Oxidative stress due to an increase in ROS production and a decrease in antioxidant defense, is well documented in doxorubicin-associated myocardial apoptosis^22,23^.

The results in this study, as well as many others, showed that administration of doxorubicin significantly increased the generation of ROS and reduced the activity of endogenous antioxidant enzymes. Intriguingly, the results clearly showed that doxorubicin-induced oxidative stress can be mitigated by the administration of grape polyphenol concentrate, which may likely be due to the antioxidant properties of polyphenols. Currently, several polyphenolic substances with antioxidant properties and cardioprotective effects have been identified, including resveratrol and epigallocatechin, which have the ability to attenuate doxorubicin-induced damage to myocardial structure and cardiac dysfunction in rats by inhibiting free radical formation, improving mitochondrial dysfunction, and suppressing apoptosis^10,19,24^.

In conclusion, the results of the current study showed that grape polyphenol concentrate, has the ability to reduce ROS production, improve antioxidant enzyme activities, and mitigates myocardial cell damage. However, we suggest that the antioxidant mechanisms of grape polyphenol concentrate is likely related to its ability to improve the activity and levels of the potent and related antioxidant enzymes, SOD, catalase and glutathione peroxidase. The grape polyphenol concentrate used in this study is a mixture of different components and thus, further studies that aim to investigate the effective components and their cardioprotective mechanisms of action are warranted.

## Materials and methods

### Polyphenol preparation

#### Grape polyphenol concentrate

Cabernet Sauvignon-old French grape variety of medium-term maturity, currently located in Almaty and Zhambyl regions of Kazakhstan, were used in the study. The concentrate polyphenols obtained from seed ridges and grape skin—secondary wine products using water-alcohol feedstock extraction (40% aqueous—alcoholic solution of ethyl alcohol in the ratio 1: 5), followed by concentrating the extract on a rotary evaporator to a dry matter content of 25%. The total concentration of phenolic derivatives in the concentrate of grape polyphenols used in this experiment is 10,000 mg/l.

### Induction of cardiotoxicity

Healthy adult *Wistar* rats aged ten to twelve weeks and weighing 200 ± 20 g were housed in the animal facility of the National Center for Biotechnology, Nur-Sultan, Kazakhstan. After one-week adaptation period, the rats were randomly divided into 2 groups (6 rats/cage) and housed in a room with controlled temperature and a 12-h light–dark cycle with unlimited access to standard food and drinking water ad libitum. Cardiomyopathy was experimentally induced by a single intraperitoneal administration of doxorubicin at 8.0 mg/kg of animal body weight. The experimental group rats were injected via intragastric route with a 0.5 ml polyphenol concentrate, which is equivalent to 25 mg of phenolic compounds per 1 kg of animal body weight. Rats in the control group, received 0.5 ml drinking water (vehicle control) within 7 days of administering polyphenol concentrate and doxorubicin injections.

### Oxidative status and biochemical analyses

In this section the hematological parameters of the different animal groups were determined using the Abacus junior vet5 hematology analyzer (DIATRON Messtechnic GmbH, Austria). The oxidative status was determined using FRAS 4 device (Evolvo S.R.L., Italy) and d-ROMs Test kits, which shows the number of free radicals. The antioxidant was measured using the Plasma Antioxidant Test kit (PAT) (H&D S.R.L., Italy), which is a photometric test that enables the determination of the total antioxidant activity of blood plasma.

### Determination of myocardial damage and antioxidant activity

#### Measurement of aspartate aminotransferase

Aspartate aminotransferase (AST) is widely distributed in the body with highest levels found in the heart and liver and its release increases from cardiomyocytes undergoing necrosis. In order to confirm the myocardial damage, the level of AST was used as a biomarker for myocardial damage that was determined using the commercially available kinetic method, AsAT-Vital kit (Vital Diagnostics SPb, Russia).

#### Determination of the level of troponin T

Another commonly used confirmatory biomarker of myocardial damage is serum troponin T (TnT), which was determined in this experiment by using ELISA kit (Cloud-Clone Corp # SEB820Ra). Briefly, the kit is a sandwich enzyme immunoassay designed for the in vitro quantitative measurement of TnT in rat serum. Freshly drawn serum allowed to clot for two hours at room temperature, centrifuged for 20 min at approximately 1,000×*g*, then placed in a 96-well plate with 100 μl of detection reagent A and then reagent B following 1 h, and then 30 min incubation at 37 °C, respectively, with washing steps in between. Finally, substrate solution was added for 30 min, followed by a stop solution and plate reading at 450 nm.

#### Measurement of catalase activity

The catalase activity assay measures the activity of the antioxidant enzyme that catalyzes the decomposition of hydrogen peroxide (H_2_O_2_), a toxic molecule to the cell, into water and oxygen, which provides protection against cellular oxidative damage. In principle, the assay measures the amount of H_2_O_2_ remaining after the action of catalase. The assay was done using serum samples that were collected by centrifuging clotted blood samples at 2,000×*g* for 15 min, and performed according to manufacturers’ guidelines. Briefly, 20 µM H_2_0_2_ per well were added to certain amounts of catalase in 1 × reaction buffer, and then incubated for 30 min at room temperature. A 100 µl sample of sodium azide was added to stop the enzymatic reaction. Aliquots were then assayed at excitation of 530 nm and emission at 590 nm to measure the amount of remaining H_2_O_2_.

#### Determination of superoxide dismutase (SOD)

The amount of SOD was determined using the SOD Determination Kit that allows the measurement of SOD in serum by utilizing the highly water-soluble tetrazolium salt, WST-1 [2-(4-Iodophenyl)-3-(4-nitrophenyl)-5-(2,4-disulfophenyl)-2H-tetrazolium, monosodium salt that produces a water-soluble formazan dye upon reduction with a superoxide anion. In summary, 20 µl of sample solution were added to a serum sample and blank-2 wells and 20 µl of ultrapure H_2_O to Bank-1 and Blank-3 wells. Then, 200 µl of WST Working Solution were added to each well, then 20 µl of Dilution Buffer were added to blank-2 and blank-3 wells, and 20 µl of Enzyme Working Solution was added to sample solution and Blank-1 wells. After a 20 min incubations at 37 °C, the absorbance was read at 450 nm. The SOD activity (inhibition rate %) was calculated using the following equation:

$$ {\text{SOD activity }}\left( {{\text{inhibition rate }}\% } \right) \, = \frac{{\left( {{\text{A}}_{{\text{Blank 1}}} {-}{\text{ A}}_{{\text{Blank 3}}} } \right) \, {-} \, \left( {{\text{A}}_{{{\text{Sample}}}} {-}{\text{ A}}_{{\text{Blank 2}}} } \right)}}{{\left( {{\text{A}}_{{\text{Blank 1}}} {-}{\text{ A}}_{{\text{Blank 3}}} } \right)}} $$

#### Measurement of glutathione level and activity

The level and activity of glutathione in the blood were measured using commercially available kits according to manufacturers’ guidelines (Sigma-CS0260 and Sigma-CS0410, respectively). Briefly, for glutathione levels, blood samples were first deproteinized with the 5% 5-sulfosalicylic acid solution and then glutathione contents were determined by kinetic assay, where the catalytic amounts of glutathione cause a continuous reduction of 5,5′-dithiobis-(2-nitrobenzoic) acid (DTNB) to TNB, which was then determined colorimetrically at 412 nm. The total activity was measured using 96-well plates and were read at 340 nm, immediately after preparing the reaction tests and every minute over a seven minutes period creating 7 time points.

### Electron microscopy analyses

Biopsy pieces of the myocardium from each animal were collected (6 rats per a group) for electron microscopy examination and analyses. The samples were fixed in a 2.5% solution of glutaraldehyde in phosphate buffer with post fixation in a 2% solution of osmium tetroxide. Semi-thin and ultra-thin sections were prepared with a Leica Ultracut UCT ultramicrotome (Leica Microsystems, Wetzlar, Germany), mounted on a slot grid covered with pioloform and contrasted with 2% uranyl acetate and lead citrate. Samples were examined, with the assistance of an experienced electron microscopy technician, on an electron microscope (LIBRA120, Carl Zeiss, Inc. Germany) with MegaView G3 TEM-camera and RADIUS imaging software. A total of 10 images per animal were captured for qualitative image analyses.

### Semiquantitative analyses of electron microscopic images

To evaluate the degree of ultrastructural damage, a semiquantitative scoring method was used. The method, which was adapted from Emir et al., and commonly used for semiquantitative analyses of electron microscope images, utilizes a four-point scale (Grade 1–4) for the morphological assessment of subcellular structures^25–28^. In brief, every slide was assessed for different morphologic changes, where “0” score designate normal and a score of “4” is given when there is a significant ultrastructural damage. The images were blindly read by histopathologists who were not aware of which animal group the images come from and assessed each criterion separately.

### Statistical analysis

All data are expressed as mean ± standard error of the mean (S.E.M.) unless stated otherwise. Data were analyzed using one-way analysis of variance (ANOVA) and Student *t*-tests. Results with *p*-values less than 0.05 (*p* < 0.05) were considered statistically significant.

### Ethics approval

Animal experiments were made in agreement with “the rules of pre-clinical studies” (approved by order of the Minister of Health and Social Development Republic of Kazakhstan on May 29, 2015 No. 415) and approved by the Ethical Committee of Nazarbayev University (Ethical approval No. 18 from 02.04.2015). The authors confirm that all experiments were performed in accordance with relevant guidelines and regulations.

## Supplementary information

Supplementary Information
