# Supplementary material for: Cardioprotective effect of grape polyphenol extract against doxorubicin induced cardiotoxicity
Source: Sci Rep. 2020 Sep 7;10:14720. doi: 10.1038/s41598-020-71827-9 (PMC7477547; doi:10.1038/s41598-020-71827-9)
Supplement: Supplementary file 1 — Supplementary Information [file 41598_2020_71827_MOESM1_ESM.docx]

Cardioprotective effect of grape polyphenol extract against doxorubicin induced cardiotoxicity.

Shynggys Sergazy^1^, Zarina Shulgau^2^, Galina Fedotovskikh^3^, Laura Chulenbayeva^1^, Ayaulym Nurgozhina^1^, Madiyar Nurgaziyev^1^, Elena Krivyh^4^, Yevgeny Kamyshanskiy^5^, Almagul Kushugulova^1^, Alexander Gulyayev^1^, Mohamad Aljofan^6,*^

**Table S1. General analyses of electron microscope**

| **Сardiomyocytes** | | | | | | | | | | | | |
| --- | --- | --- | --- | --- | --- | --- | --- | --- | --- | --- | --- | --- |
| **Grade\ organelle** | **MT [1]** | **M and Zl [1]** | **C and N [1]** | **Gl [2]** | **ngGl** | **IE** | **SE** | **IV [3]** | **GER[3]** | **SER [3]** | | **MM** |
| **1** | Unchang; electron-dense matrix with granules and densely packed cristae | Myofibril structure and Z-line | Round/oval shape, intact nucleolus, homogeneous chromatin, and continuous basal lamina | 31 ηm -42 ηm | Small | Normal | Normal | Normal | Normal | Normal | | Normal |
| **2** | Matrix locally electron lucent; loss of granules | Wavy Z-line | Change in shape | 21 ηm -30 ηm | Middle | Light edema | Light edema | Small | Dilated | Dilated in places | | Small |
| **3** | Swelling and/or fragmentation of cristae | Blurring, breakage, thickening of Z-line | Convoluted nuclear membrane | 11 ηm-20 ηm | Large | Mild edema | Mild edema | Middle | Corrupted lamellar  arrangement | Existing vacuoles | | Middle |
| **4** | Presence of concentric cristae | No myofibril structure | Rupture of cell membrane and/or karyolysis | <10 ηm | - | Severe edema | Severe edema | Large | Broken | Fields of broad degeneration  þ myelin figures | | Large |
| **Еndothelium** | | | | | | | | | **BM** | | |  |
| **Grade\ organelle** | **N [3]** | **MT [3]** | **EC [4]** | **IE [4]** | **SE** | **IV [3]** | **GER[3]** | **SER [3]** | **eBM** | | **tBM** | |
| **1** | Normal | Normal | Slight thinning | Normal | Normal | Normal | Normal | Normal | Normal | | Normal | |
| **2** | Disintegrated chromatin  (margination, clumping) | Clear cristae | Apparent thinning | Light edema | Light edema | Small | Dilated | Dilated in places | Light edema | | Slight thinning | |
| **3** | Increased hetero-chromatin | Oedematous | Detachment from basement membrane | Mild edema | Mild edema | Middle | Corrupted lamellar  arrangement | Existing vacuoles | Mild edema | | Apparent thinning | |
| **4** | Degenerated nucleuses | Accumulation of amorphous material | Detachment from basement membrane and apparent thinning | Severe edema | Severe edema | Large | Broken | Fields of broad degeneration  þ myelin figures | Severe edema | | - | |

**Table S2. Semi quantification of electron microscope analyses**

| **Сardiomyocytes** | | | | | | | | | | | |
| --- | --- | --- | --- | --- | --- | --- | --- | --- | --- | --- | --- |
| **Grade\ organelle** | **MT [1]** | **M and Zl [1]** | **C and N [1]** | **Gl [2]** | **ngGl** | **IE** | **SE** | **IV [3]** | **GER[3]** | **SER [3]** | **MM** |
| **Doxorubicin**  **(n-6)** | 2.3±1.0 | 2.8±0.8 | 3.0±2.9 | 2.8±0.8 | 2.8±0.4 | 2.2±0.8 | 2.2±0.4 | 1.8±1.0 | 1.5±0.5 | 1.3±0.5 | 2.0±0.9 |
| **Doxorubicin and grape polyphenol concentrate**  **(n-6)** | 2.2±0.4 | 1.3±0.5 | 2.2±1.2 | 1.3±0.5 | 1.2±0.4 | 2.2±0.4 | 0.0±0.0 | 1.5±0.8 | 1.2±0.4 | 1.2±0.4 | 1.8±0.8 |
| **P-value** | 0.937 | **0.015*** | 0.240 | **0.009*** | **0.002*** | 0.937 | **0.000*** | 0.589 | 0.394 | 0.699 | 0.818 |
| **Еndothelium** | | | | | | | | | **BM** | |  |
| **Grade\ organelle** | **N [3]** | **MT [3]** | **EC [4]** | **IE [4]** | **SE** | **IV [3]** | **GER[3]** | **SER [3]** | **eBM** | **tBM** | |
| **Doxorubicin**  **(n-6)** | 2.3±1.0 | 1.7±0.8 | 1.8±0.8 | 2.0±0.6 | 2.0±0.6 | 2.0±0.6 | 2.0±0.6 | 1.7±0.5 | 2.2±0.8 | 2.2±0.8 | |
| **Doxorubicin and grape polyphenol concentrate**  **(n-6)** | 2.2±0.4 | 1.5±0.8 | 1.3±0.5 | 1.8±0.4 | 1.0±0.0 | 1.8±0.4 | 1.8±0.4 | 1.7±0.8 | 1.2±0.4 | 1.2±0.4 | |
| **P-value** | 0.937 | 0.699 | 0.310 | 0.699 | **0.000*** | 0.485 | 0.699 | 0.937 | **0.041*** | **0.026*** | |

MT- mitochondria, M and Zl - Myofibril structure and Z-line, C and N - Cytoplasm and nucleus, Gl - glycogen, ngGl - number of glycogen granules, IE- Intracytoplasmic edema, SE- subsarcolem edema, GER - granulated endoplasmic reticulum, SER - smooth endoplasmic reticulum, MM- myelination of the membranes EC- endothelial cells, IV - intracytoplasmic vacuoles, BM - basement membrane, eBM – BM edema, tBM - thickness BM.

* P-value <0.05
